# Combinatorial multivalent interactions drive cooperative assembly of the COPII coat

**DOI:** 10.1083/jcb.202007135

**Published:** 2020-09-30

**Authors:** Viktoriya G. Stancheva, Xiao-Han Li, Joshua Hutchings, Natalia Gomez-Navarro, Balaji Santhanam, M. Madan Babu, Giulia Zanetti, Elizabeth A. Miller

**Affiliations:** 1 MRC Laboratory of Molecular Biology, Cambridge, UK; 2 Institute of Structural and Molecular Biology, Birkbeck College, London, UK

## Abstract

Protein secretion is initiated at the endoplasmic reticulum by the COPII coat, which self-assembles to form vesicles. Here, we examine the mechanisms by which a cargo-bound inner coat layer recruits and is organized by an outer scaffolding layer to drive local assembly of a stable structure rigid enough to enforce membrane curvature. An intrinsically disordered region in the outer coat protein, Sec31, drives binding with an inner coat layer via multiple distinct interfaces, including a newly defined charge-based interaction. These interfaces combinatorially reinforce each other, suggesting coat oligomerization is driven by the cumulative effects of multivalent interactions. The Sec31 disordered region could be replaced by evolutionarily distant sequences, suggesting plasticity in the binding interfaces. Such a multimodal assembly platform provides an explanation for how cells build a powerful yet transient scaffold to direct vesicle traffic.

## Teaser

Using in vitro reconstitution assays and in vivo cellular phenotypes, Stancheva and colleagues dissect the protein interaction network that drives COPII coat assembly during vesicle formation from the endoplasmic reticulum, revealing the importance of multivalent interactions that mutually reinforce each other.

## Introduction

Proteins within the secretory pathway are transported by vesicles generated by cytoplasmic coat proteins, which simultaneously recruit appropriate cargo and sculpt the donor membrane into spherical structures. Vesicle formation requires significant force to overcome the intrinsic rigidity of the lipid bilayer, and coat proteins solve this problem by organizing into oligomeric scaffolds that can impose structure on the underlying membrane (Stachowiak et al., 2013; Derganc et al., 2013). The COPII coat comprises five proteins that self-assemble on the cytosolic face of the ER membrane to traffic nascent secretory proteins toward the Golgi. COPII assembly is initiated upon GTP binding by the small GTPase Sar1, which exposes an amphipathic α-helix that embeds shallowly in the membrane (Hutchings et al., 2018). GTP-bound Sar1 recruits Sec23–Sec24 (Matsuoka et al., 1998), which is the cargo-binding subunit of the coat (Mossessova et al., 2003; Miller et al., 2002). The Sar1–Sec23–Sec24 “inner coat” complex in turn recruits the “outer coat,” Sec13–Sec31, to drive vesicle formation (Antonny et al., 2001; Matsuoka et al., 1998). Sec13–Sec31 tetramers form rods (Fath et al., 2007) that can self-assemble in vitro into a cage-like structure that closely matches the size and geometry of vesicles observed by EM (Stagg et al., 2006). Sar1–Sec23–Sec24 can also form higher order assemblies (Zanetti et al., 2013), but Sec13–Sec31 is required to organize these arrays (Hutchings et al., 2018).

The GTP hydrolysis cycle is key to coat assembly and disassembly, thereby creating a dynamically metastable structure. Sar1 alone has low intrinsic GTPase activity, and requires its GTPase-activating protein, Sec23, for GTP hydrolysis (Yoshihisa et al., 1993). Sec31 further accelerates this reaction (Antonny et al., 2001) via a short “active fragment” that contacts both Sec23 and Sar1 (Bi et al., 2007). Since GTP hydrolysis triggers coat disassembly (Barlowe et al., 1994; Antonny et al., 2001), the maximal GTPase activity induced by recruitment of the outer coat creates a paradox. How is coat assembly stabilized to counter the destabilizing effect of GTP hydrolysis and prolong coat association sufficiently to produce a vesicle? First, the presence of cargo proteins prolongs coat association with the membrane after GTP hydrolysis (Hughes and Stephens, 2008; Sato and Nakano, 2005; Iwasaki et al., 2017). Another mechanism that likely promotes coat stability is the modulation of outer coat recruitment by regulatory factors, like Sec16 and TANGO1, that compete for binding to Sec23–Sec24–Sar1 (Yorimitsu and Sato, 2012; Kung et al., 2012; Saito et al., 2009; Ma and Goldberg, 2016). Finally, the inherent instability of the full coat upon GTP hydrolysis is likely countered by additional stabilizing interactions between the inner and outer coat layers (Ma and Goldberg, 2016; Hutchings et al., 2018). To fully understand the interplay between coat dynamics and stability, deeper insight into the interfaces that drive the coat to its oligomeric state is required.

A long, unstructured proline-rich domain of Sec31 mediates the only known means of interaction between the inner and outer coat (Bi et al., 2007; Ma and Goldberg, 2016). The first interface involves the active fragment, which encompasses ∼50 residues of this domain. It occupies an extended surface on the inner coat with two key residues, W_922_ and N_923_ in *Saccharomyces cerevisiae* Sec31, inserted into the Sar1•Sec23 active site (Bi et al., 2007; Hutchings et al., 2018). The functional importance of the active fragment interface is demonstrated by the F382L mutation in human Sec23A. This substitution occurs in the region of Sec23 bound to the active fragment and causes musculoskeletal defects (Boyadjiev et al., 2006; Fromme et al., 2007). When paired with Sar1B, the F382L mutation prevents in vitro recruitment of the outer coat and impairs budding. However, patient cells retain frustrated membrane budding profiles at ER exit sites, suggesting some membrane remodeling can still occur (Fromme et al., 2007).

The second known inner-outer coat interface is less well studied, and involves binding of triple-proline (PPP) motifs to the gelsolin domain of Sec23 (Hutchings et al., 2018; Ma and Goldberg, 2016). This interaction was initially identified for PPP motifs on the procollagen export receptors, TANGO1 and cTAGE5, each of which contains multiple PPP motifs within their unstructured proline-rich domains (Ma and Goldberg, 2016). The multivalent nature of these interaction motifs was speculated to promote local recruitment of Sar1–Sec23–Sec24 and template a helical arrangement of the inner coat compatible with formation of a coated tubule rather than a spherical bud (Hutchings et al., 2018; Ma and Goldberg, 2016). Sec31 also has multiple PPP motifs and competes for the TANGO1–Sec23 interaction, perhaps thereby displacing TANGO1 and completing coat assembly (Ma and Goldberg, 2016; Saito et al., 2009). Electron density that likely corresponds to the Sec23–Sec31 gelsolin–PPP interface is clearly observed in liposome tubules coated with yeast COPII proteins, confirming that this interface is a relatively stable part of the assembled coat (Hutchings et al., 2018). TANGO1 appears specific to the metazoan lineage (Klute et al., 2011), but PPP motifs are also found in the conserved regulatory protein Sec16, suggesting a more ancient origin for coat assembly by PPP recognition. The functional importance of PPP-mediated interactions in full coat assembly, and in cells, remains to be tested.

Here, we aimed to obtain a more complete picture of the interactions that drive coat assembly by testing the functional importance of interfaces that contribute to outer coat oligomerization and recruitment to the inner coat layer. We combine genetic perturbation with in vitro reconstitution assays to test the essentiality of individual interactions and determine how specific mutants are defective. We find that coat assembly is driven by a combination of evolutionarily conserved sequence features that create a multivalent interface between the inner and outer coat layers. These interactions counter the instability associated with GTP hydrolysis to promote productive vesicle formation. Moreover, outer coat cage assembly via a known Sec31–Sec31 structural interface reinforces these interactions, suggesting a feed-forward mechanism for coat propagation. Finally, we show that diverse protein sequences that preserve both global and local sequence elements can suffice for coat assembly and viability.

## Results

### PPP-driven interactions are dispensable for coat assembly but contribute to coat stability

**Figure 1.**
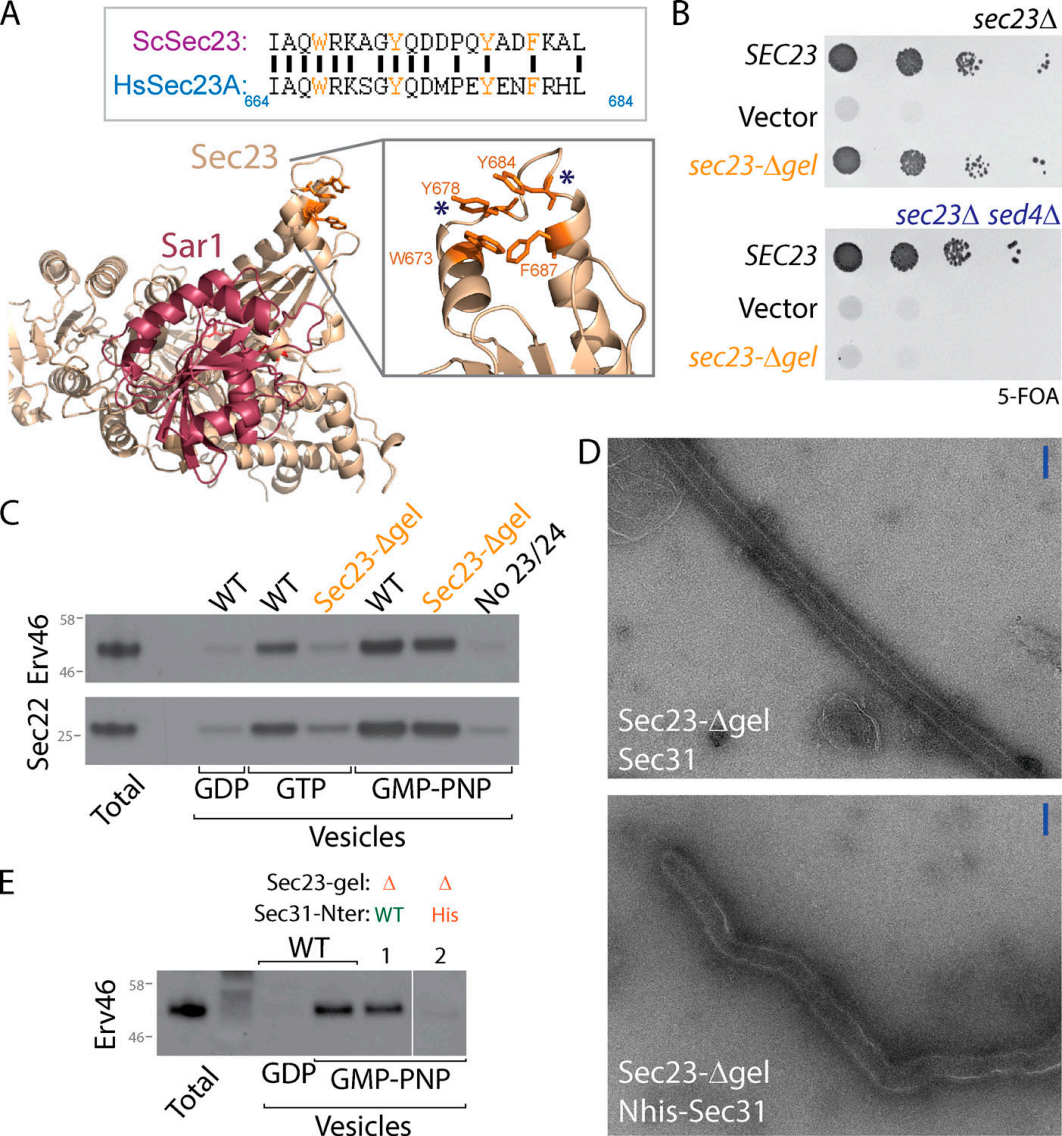
**Sec23 gelsolin domain is dispensable for coat assembly but contributes to coat stability. (A)** Structural model (PDB accession no. 1M2O) of Sar1 (red) and Sec23 (wheat) highlighting the membrane-distal gelsolin domain (inset) that contains conserved aromatic residues (orange) that in human Sec23 contribute to PPP binding. The loop between the asterisks was replaced with a glycine-serine-glycine linker to generate *sec23-Δgel.* **(B)** *sec23Δ* and *sec23Δ sed4Δ* strains were transformed with the indicated plasmids and grown on media containing 5-FOA, which counterselects for a *SEC23::URA* plasmid. Growth on 5-FOA is indicative of ability to serve as the sole copy of *SEC23*. Deletion of the gelsolin loop was tolerated in the *sec23Δ* strain but not in the *sec23Δ sed4Δ* double mutant. **(C)** In vitro budding experiments using yeast microsomal membranes incubated with Sar1, Sec23–Sec24, Sec13–Sec31, and the indicated nucleotides. Vesicle release from the donor membrane (Total) is measured by detecting incorporation of cargo proteins, Sec22 and Erv46, into a slowly sedimenting vesicle fraction. When WT Sec23 was replaced with Sec23-Δgel, budding was compromised only in the GTP condition. **(D)** Negative-stain electron microscopy of GUVs incubated with the indicated WT or mutant proteins. Scale bars, 100 nm. **(E)** In vitro budding as described for C, but where one reaction used Sec31 that was tagged with hexahistidine at the N-terminus (lane 2). Sec23-Δgel in combination with Nhis-Sec31 was compromised for budding even in the presence of GMP-PNP.

We first tested the importance of PPP motifs in COPII assembly by mutagenesis of the Sec23 gelsolin domain that interacts with PPP motifs. Four aromatic residues that form the PPP-binding cleft are conserved between yeast and humans (Fig. 1 A), and mutation of these residues abrogated PPP binding to human Sec23A (Ma and Goldberg, 2016). We engineered a mutant that replaced key hydrophobic residues in the PPP-binding cleft with a glycine-serine-glycine tripeptide. This gelsolin loop mutant (*sec23-Δgel*) complemented a *sec23Δ* null strain, revealing that PPP binding is not essential for coat function (Fig. 1 B). We reasoned that this interaction might become more important under conditions of dynamic coat turnover. We therefore tested whether perturbations to the GTPase cycle of the coat might sensitize yeast to the loss of the PPP-binding interface. Sed4 is a nonessential accessory factor that is thought to assist Sec16 in Sar1 GTP regulation (Kung et al., 2012; Gimeno et al., 1995). Indeed, *sec23-Δgel* was inviable when *SED4* was also deleted, suggesting that compromised PPP-binding by Sec23 becomes problematic when the GTP cycle of the coat is altered (Fig. 1 B). To gain further insight into the nature of the defect associated with perturbation of the gelsolin domain, we used an in vitro assay that reconstitutes vesicle formation from purified microsomal membranes (Barlowe et al., 1994). Vesicle formation following incubation with purified COPII proteins is monitored by the presence of cargo proteins (Erv46 and Sec22) in a slowly sedimenting vesicle fraction. This budding assay showed that Sec23-Δgel could drive vesicle formation with a non-hydrolyzable GTP analogue, GMP-PNP, but not with GTP (Fig. 1 C). Thus, when coat assembly is stabilized by inhibiton of GTP hydrolysis, perturbation of the gelsolin-PPP interaction has minimal effect, but under the condition of GTP-dependent coat turnover (Antonny et al., 2001), loss of this interface impairs vesicle formation. These findings support the model that Sec23–Sec31 interactions help stabilize the assembling coat to counter instability triggered by GTP hydrolysis.

**Figure S1.**
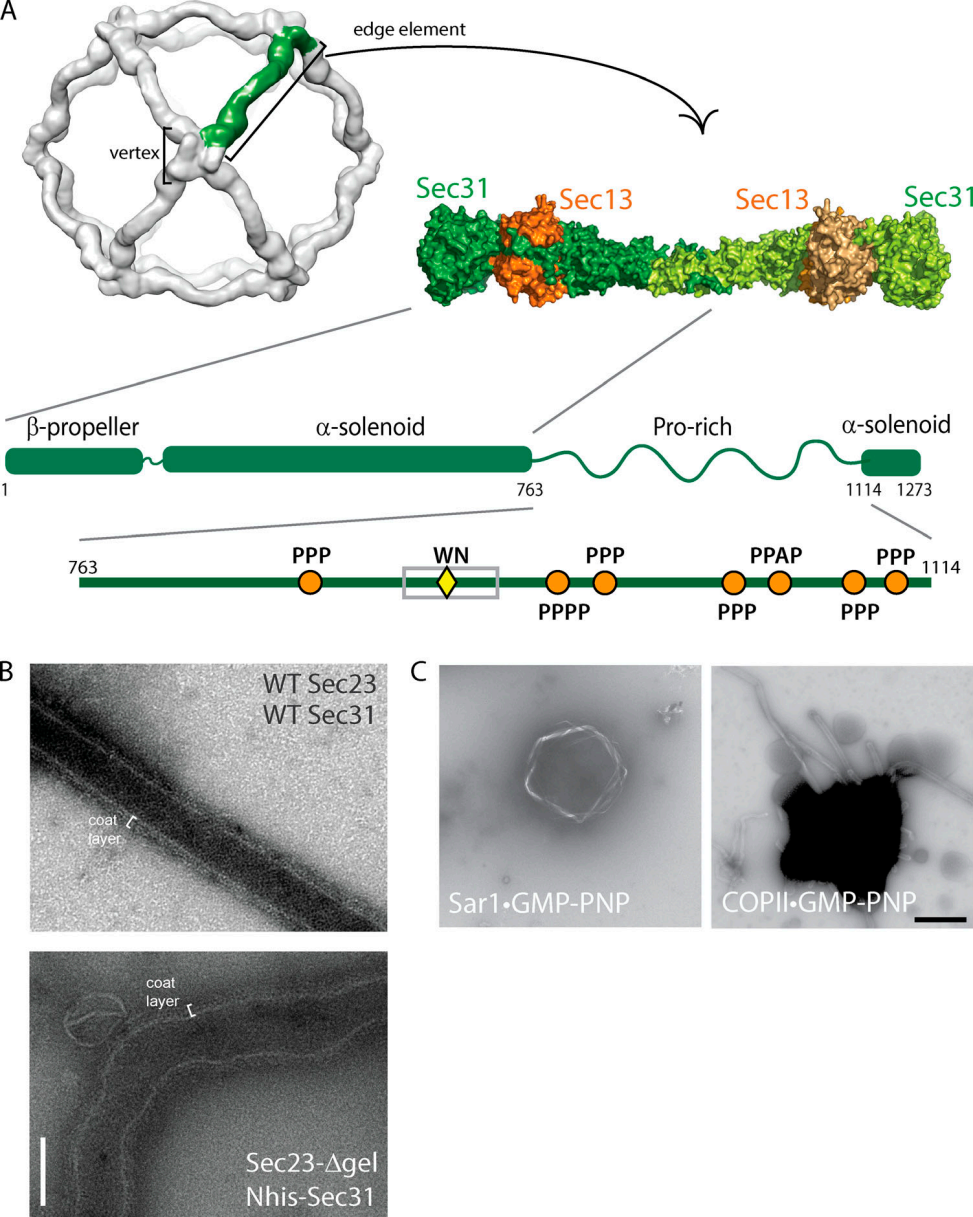
**Structure and assembly of the COPII outer coat. (A)** Cryoelectron microscopy model of the cage structure formed by mammalian Sec13–Sec31 (EMD-5524; Noble et al., 2013), showing the “cage vertex” where Sec31 β-propeller domains tetramerize, and crystal structure (PDB accession nos. 2PM6 and 2PM9; Fath et al., 2007) of the yeast Sec13–Sec31 “edge element” that forms the assembly unit. **(B)** Negative-stain electron microscopy of GUVs incubated with Sar1•GMP-PNP and the indicated WT or mutant proteins. Scale bar, 100 nm. **(C)** Low-magnification negative-stain electron microscopy of GUVs incubated with Sar1•GMP-PNP alone (left) or the full COPII coat (right) shows dependence on the full coat for tubulation. Scale bar, 1 µm.

A GTP-specific vesicle budding defect was also observed with N-terminally His-tagged Sec31 (Hutchings et al., 2018), where the histidine tag is thought to interfere with interactions that drive assembly of the cage “vertex” (Fig. S1 A). Thus, in the context of coat turnover promoted by GTP hydrolysis, destabilization of interfaces that drive either outer coat oligomerization or inner/outer coat assembly is incompatible with coat function in vitro. We therefore tested whether loss of both interfaces would perturb coat function using a membrane-bending assay that measures tubulation of giant unilamellar vesicles (GUVs) in the presence of GMP-PNP. When combined with WT Sec31, Sec23-Δgel yielded straight lattice-coated tubules similar to WT (Figs. 1 D and S1 B). However, when GUV tubulation was induced using Sec23-Δgel and Nhis-Sec31, tubes were irregular, and although a coat layer was visible, no extended coat lattice was seen (Figs. 1 D and S1 B). We note that high concentrations of Sar1 alone can induce membrane tubulation (Lee et al., 2005), but the conditions we use require the full COPII coat to yield tubules (Fig. S1 C). Nonetheless, when both outer coat oligomerization and inner/outer coat interactions are reduced, residual membrane curvature is likely driven by coat features such as the Sar1 α-helix and inner coat oligomerization (Hutchings et al., 2018; Lee et al., 2005).

Consistent with less robust residual membrane remodeling associated with loss of multiple coat interfaces, in vitro vesicle budding from microsomal membranes was not supported by Sec23-Δgel combined with Nhis-Sec31, (Fig. 1 E). One interpretation of these findings is that two separate interactions, Sec23–Sec31 binding via PPP motifs and cage assembly via Sec31–Sec31 vertex interfaces, mutually reinforce each other to propagate coat stability. This combinatorial interaction is especially important in the context of a cargo-replete membrane, which likely resists the membrane bending force of the coat (Copic et al., 2012). Together, our Sec23 mutagenesis experiments suggest that PPP binding contributes to, but is not essential for, coat assembly. The importance of the PPP interaction is revealed when the GTP cycle of the coat is altered, suggesting that the nucleotide-associated interface and PPP binding are mutually productive in COPII assembly.

**Figure 2.**
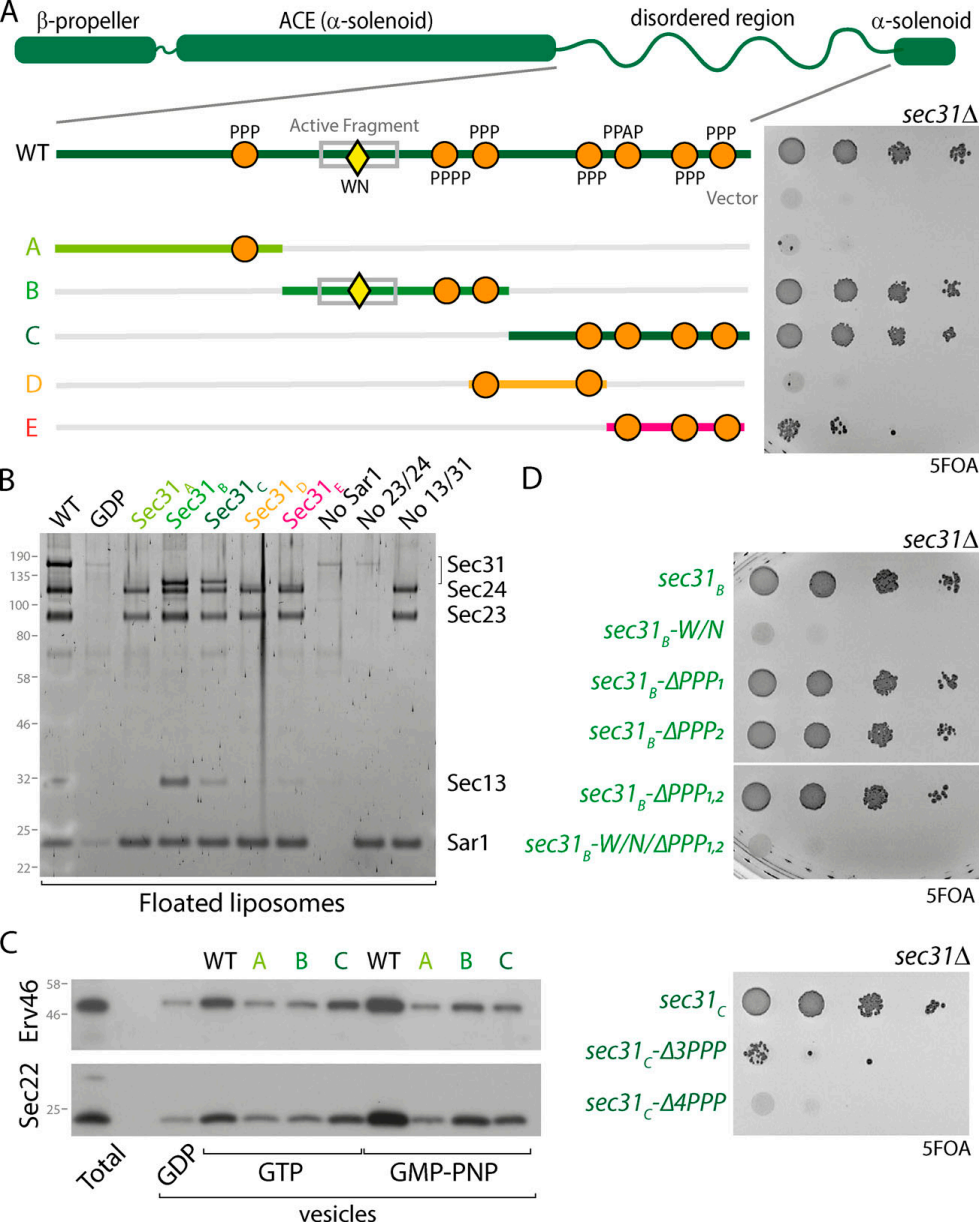
**The Sec31 disordered region contain multiple independent drivers of assembly. (A)** Diagram of Sec31 showing structured elements (β-propeller and α-solenoids) and the proline-rich disordered region that contains the active fragment (gray box) with GTP stimulatory residues W_922_/N_923_ residues (yellow diamond) and PPP motifs (orange circles). The disordered region of Sec31 was dissected into the indicated fragments, and the ability of these shortened regions to function in place of the full-length disordered region was tested by serial dilution on 5-FOA. Only the B and C fragments support viability. **(B)** Purified Sar1, Sec23–Sec24, and Sec13–Sec31 were incubated with synthetic liposomes in the presence of GMP-PNP and the liposomes purified by flotation through a sucrose gradient. Bound material (floated liposomes) was collected and examined by SDS-PAGE to visualize coat proteins. Recruitment of proteins with shortened disordered regions to liposomes correlates with viability: only B and C fragments were recruited. **(C)** In vitro budding from microsomal membranes with the indicated proteins also correlated with viability but showed nucleotide dependence. **(D)** Serial dilutions of Sec31_B_ and Sec31_C_ after mutation of the indicated binding elements reveals the importance of these sequences in the context of a shorter disordered region.

**Figure S2.**
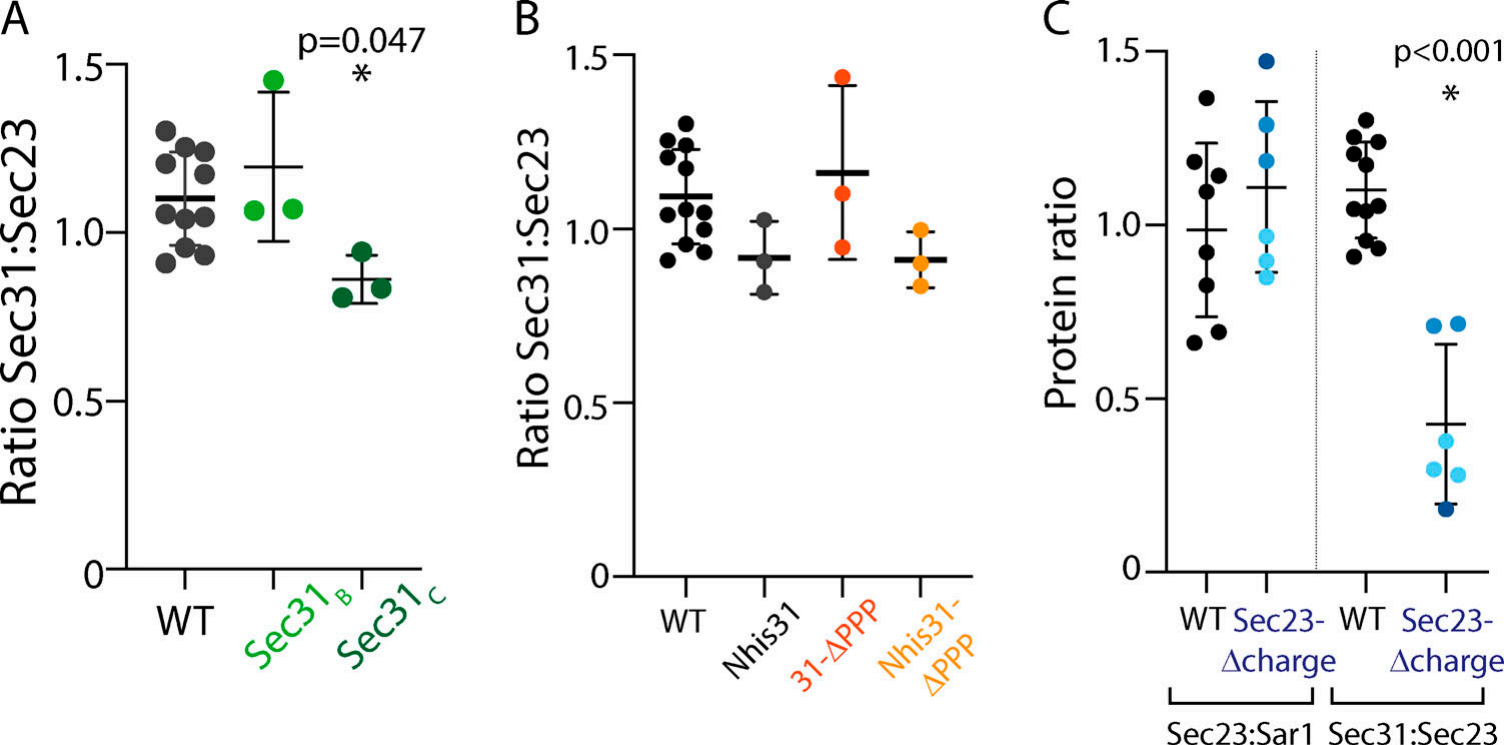
**Quantification of liposome recruitment assays. (A)** The ratio of Sec31 to Sec23 was quantified from multiple independent liposome recruitment experiments (see Fig. 2 B) using the indicated proteins, revealing a slight deficit in recruitment of Sec31_C_. **(B)** The ratio of Sec31 to Sec23 was quantified from multiple independent experiments (see Fig. 3 C) using the indicated proteins, revealing no difference in recruitment. **(C)** Ratios of Sec23 to Sar1 (left values) and Sec31 to Sec23 (right values) were calculated from multiple independent experiments (see Fig. 4 D) revealing that Sec23-Δgel was recruited like WT protein to Sar1 but was impaired in its ability to recruit Sec31. In each case, error bars depict standard deviation, and the statistical test was a one-way ANOVA with Dunnett’s multiple comparisons test. If no P value is given, then there was no difference.

We sought to further probe inner/outer coat interactions by dissecting the contribution of the different Sec23-binding elements within Sec31. We divided the Sec31 proline-rich region into three segments of equivalent length and generated constructs that preserved the structural domains (the N-terminal β-propeller that forms the cage vertex, the α-solenoid rods, and a C-terminal domain of predicted α-helical structure) but replaced the disordered region with shortened fragments. Each segment retained at least one PPP motif (Fig. 2 A). The first third (*sec31_A_*), with a single PPP motif, was unable to support viability, whereas each of the two subsequent segments (*sec31_B_* and *sec31_C_*) complemented a *sec31Δ* null strain (Fig. 2 A). We further dissected the C-terminal portion of the disordered region into two smaller fragments, each of which contained at least two PPP motifs, but neither supported viability (*sec31_D_* and *sec31_E_*; Fig. 2 A). We tested each of the shortened constructs in a coat recruitment assay that measures binding of purified proteins to small (400 nm) unilamellar liposomes in the presence of GMP-PNP. Shortened proteins that conferred viability supported coat recruitment, although Sec31_C_ bound less robustly than Sec31_B_, suggesting reduced affinity (Figs. 2 B and S2 A). In contrast, the proteins that were not viable in cells were not recruited to liposomes, suggesting a critical loss in binding affinity. In the in vitro budding assay, we saw distinct effects for Sec31_B_ and Sec31_C_ (Fig. 2 C). Budding reactions with GTP were supported by Sec31_C_, but not Sec31_B_, consistent with the active fragment within Sec31_B_ stimulating GTP hydrolysis and thereby destabilizing the coat prematurely in the absence of additional stabilizing PPP motifs. Conversely, in the presence of GMP-PNP, Sec31_B_ supported vesicle release whereas Sec31_C_ was less effective. Again, this is consistent with the interface occupied by the active fragment, where the Sec23–Sar1•GMP-PNP complex would stabilize association with Sec31_B_, prolonging interaction to drive vesicle formation.

We mutagenized the interaction surfaces in Sec31_B_ and Sec31_C_ to quantify the contributions of each binding element. We made point mutations in the active fragment region, changing W_923_ and N_923_ to alanine (termed the WN mutation). Density corresponding to these residues is clearly detected in the assembled coat visualized by electron cryotomography (Hutchings et al., 2018), suggesting this is a particularly stable part of the active fragment interface. The *sec31_B_-WN* mutant did not support viability, whereas glycine/serine substitutions of the PPP motifs, either individually or in combination, was tolerated (Fig. 2 D). In contrast, mutation of the PPP motifs in Sec31_C_ abrogated viability (Fig. 2 D). These growth phenotypes are consistent with the in vitro budding phenotypes. The active fragment is the dominant interface for the B-fragment, rendering its functionality sensitive to nucleotide. In contrast, the PPP motifs drive interaction for the C-fragment, which is independent of nucleotide state but less efficient in vitro, perhaps because of reduced overall affinity. Finally, the inviability of the shortest fragments, which retain multiple PPP motifs, may reflect a requirement for a minimum length of disordered region to span the inner/outer coat distance and perhaps bridge adjacent inner coat subunits. Together, the phenotypes of these constructs with shortened disordered regions demonstrate the importance of the active fragment and PPP motifs in isolation and confirm that multiple interfaces combine to drive robust assembly.

**Figure 3.**
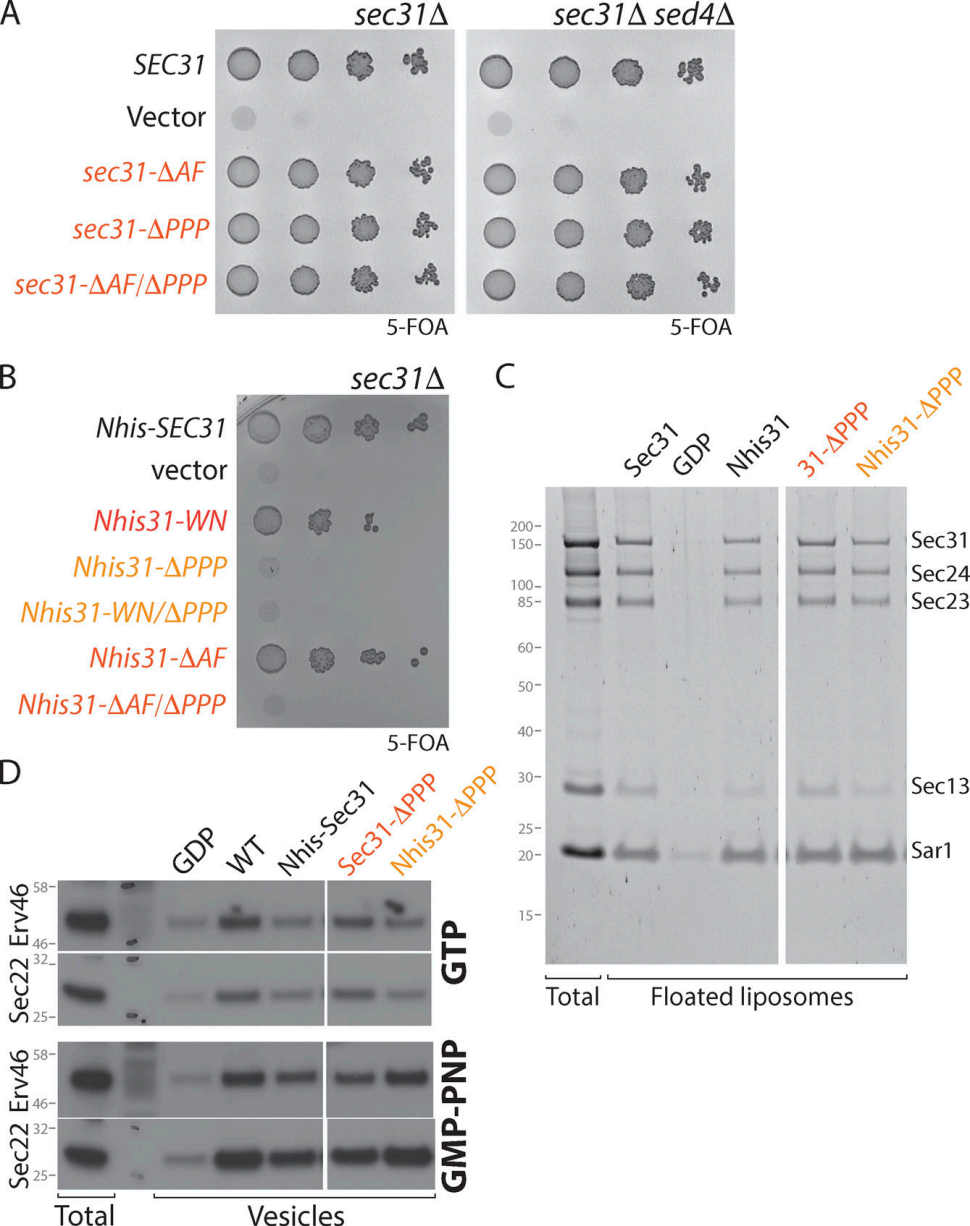
**Sec23–Sec31 interaction interfaces are dispensable for coat assembly. (A)** Serial dilutions of *sec31Δ* and *sec31Δ sed4Δ* strains expressing the deletion and substitution mutants indicated were grown on 5-FOA, revealing no loss of fitness associated with the loss of any of these interfaces. **(B)** Serial dilutions of a *sec31Δ* strain expressing Nhis-Sec31 mutants as indicated were grown on 5-FOA, revealing lethality associated with the combination of the N-terminal His tag and loss of PPP motifs. **(C)** Recruitment of the coat to synthetic liposomes as described for Fig. 2 B revealed that each of the mutants indicated were recruited as well as WT. **(D)** In vitro budding from yeast microsomes with the proteins indicated revealed normal budding efficiency, except for the Nhis-tagged proteins, which were reduced in their ability to generate vesicles with GTP.

Having demonstrated the importance of individual inner coat interaction interfaces in the context of a shortened disordered region, we next tested their relevance in the context of full-length Sec31. Neither complete deletion of the active fragment nor glycine/serine substitution of all six PPP motifs, plus an additional PPAP motif that might also bind to the gelsolin domain (Ma and Goldberg, 2016; *sec31-ΔPPP*), caused any growth defects, either alone or in combination (Fig. 3 A). Moreover, unlike the Sec23 interface mutant, deletion of *SED4* did not cause synthetic lethality with Sec31 mutants (Fig. 3 A). Thus, Sec23 mutations seem to render the coat more susceptible to GTP cycle perturbation, perhaps because they also abrogate interaction with PPP motifs on Sec16, which may modulate the GTP cycle (Kung et al., 2012; Yorimitsu and Sato, 2012). We next tested the effect of Sec31 interface mutations in the context of Nhis-Sec31, where cage assembly is perturbed. In this context, loss of the PPP motifs was lethal, whereas the W_922_A/N_923_A mutation and complete deletion of the active fragment was tolerated (Fig. 3 B). In the liposome binding assay, Nhis-Sec31-ΔPPP was recruited normally (Figs. 3 C and S2 B) and could support vesicle formation in the presence of GMP-PNP (Fig. 3 D). Thus, when the coat is stabilized by a non-hydrolyzable GTP analogue, impairment of multiple coat interfaces is tolerated, but under conditions of GTP-mediated coat turnover, combinatorial loss of these interactions is lethal. Together, mutagenesis of the known inner/outer coat interfaces supports the model that multiple coat interactions cooperatively stabilize the assembly pathway to counter the destabilizing effects of GTP hydrolysis and reveals that these interactions do not explain the entirety of coat assembly.

### A charge-driven interaction interface contributes to coat assembly

**Figure 4.**
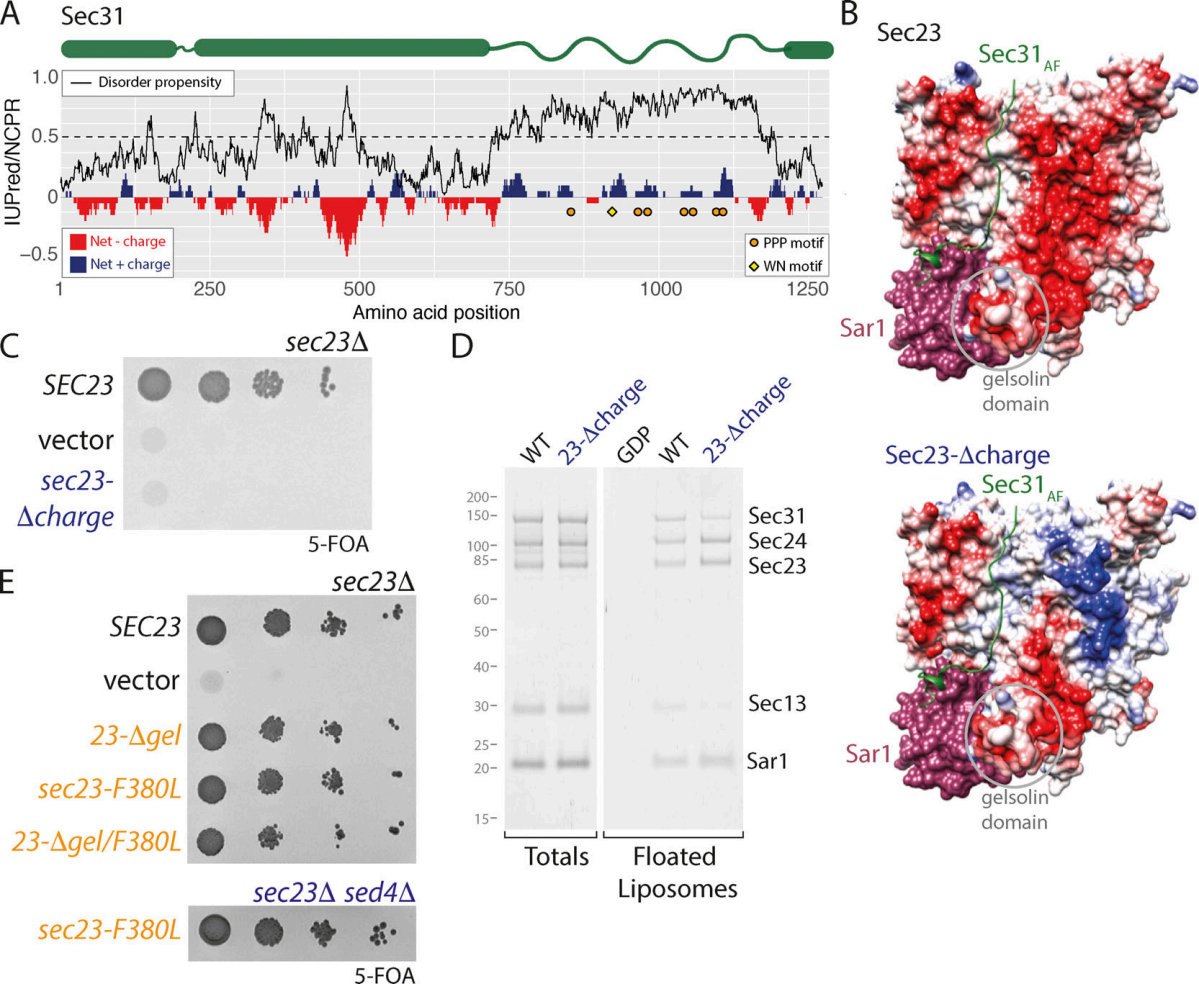
**Charge interactions contribute to assembly. (A)** Charge/disorder plot for Sec31. The black curve indicates predicted disorder propensity (IUPred); a value >0.5 (dashed line) suggests high propensity for being intrinsically disordered. Red/blue bars correspond to net charge per residue (NCPR) in a sliding window of 20 amino acids. PPP motifs are indicated by orange circles; the WN motif is indicated by yellow diamond. **(B)** Surface rendering of the crystal structures of Sar1 (pink) and Sec23 (colored according to electrostatic potential) bound to the active fragment (AF) of Sec31 (green), highlighting a negatively charged patch (red region) on the membrane-distal face, which lies to the right of the active fragment (upper panel). Substitutions in this region change the electrostatic potential in the Sec23-Δcharge mutant (lower panel). **(C)** Serial dilutions of *sec23Δ* strains transformed with the plasmids indicated reveals lethality of charge reversal of the negative patch. **(D)** Liposome flotation using the indicated proteins; recruitment of Sec13/Sec31 to liposomes coated with the Sec23-Δcharge mutant is reduced relative to WT. **(E)** Serial dilutions of *sec23Δ* or *sec23Δ sed4Δ* strains transformed with the plasmids indicated reveals no growth defects associated with the F380L mutation.

**Figure S3.**
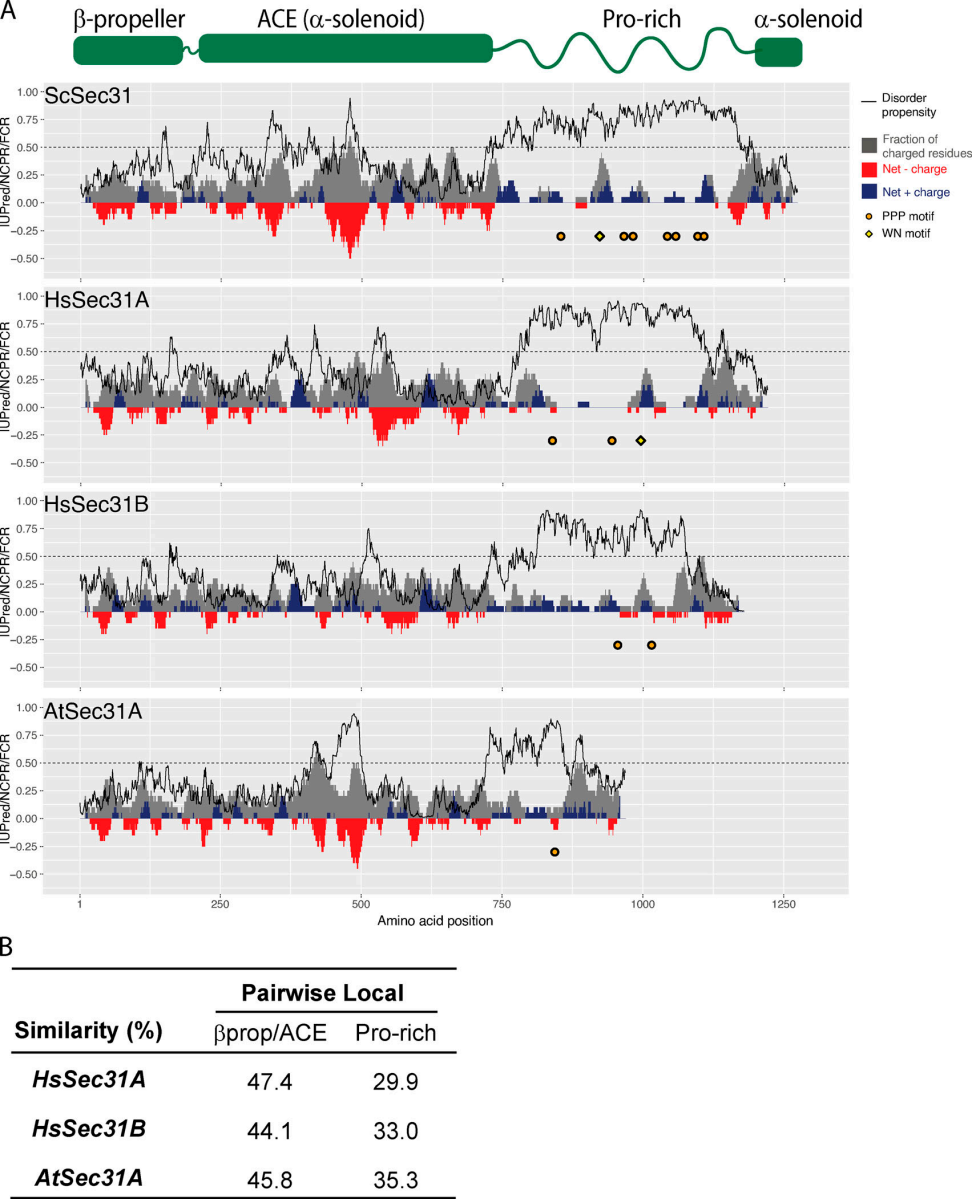
**Sec31 disordered domains share several features across species. (A)** Charge-disorder plots for Sec31 orthologues from *S. cerevisiae* (ScSec31), human (HsSec31A and HsSec31B), and *A*.* thaliana* (AtSec31A). The black curve indicates predicted disorder propensity as calculated by IUPred. A value of IUPred >0.5 (dashed line) suggests a strong propensity for being intrinsically disordered. Each gray bar corresponds to fraction of charged residues (FCR) in a sliding window of 20 amino acids, centered on the residue indicated. Red/blue bars at each position correspond to net charge per residue (NCPR) in a sliding window of 20 amino acids. PPP motifs are indicated by orange circles, and WN motifs are indicated by yellow diamonds. **(B)** Local pairwise sequence similarity between the ScSec31 structural domain (βprop/ACE) and disordered regions (Pro-rich) and corresponding domains in orthologues. None of the orthologues share significant similarity to ScSec31 within their disordered regions, whereas the structured regions have higher homology.

The minimal phenotypic consequences of ablating the known interactions between coat layers (PPP plus active fragment; Fig. 3 A) suggests that additional interfaces contribute to coat assembly. We examined the Sec31 proline-rich disordered region for conserved features that might indicate functional importance (van der Lee et al., 2014), focusing on charge properties. We found clusters of charged residues across different regions of Sec31, including regions of net negatively charged residues in the structured domain, and smaller clusters of net positive charges in the disordered region (Fig. 4 A). Positive charge clusters were also features of Sec31 orthologues in human and *Arabidopsis thaliana* (Fig. S3 A). We searched for a corresponding surface on Sec23 that may comprise a charge-driven interface. We identified a negatively charged surface adjacent to the gelsolin domain, separate from that occupied by the active fragment (Fig. 4 B, top panel). Reversing the charges of this region on Sec23 (Fig. 4 B, bottom panel) led to lethality (Fig. 4 C). The mutant protein was stable and readily purified, and it was defective for recruitment of Sec13–Sec31 in the liposome binding assay (Figs. 4 D and S2 C). We propose that charge-driven interactions between Sec23 and Sec31 represent a novel third binding mode that contributes to inner/outer coat interactions. Indeed, recent structural analysis reveals that this region of Sec23 participates in a protein–protein interaction in the context of the fully assembled coat, consistent with our proposal that positively charged regions of Sec31 engage this site (Hutchings et al., 2020 *Preprint*).

Given the profound effect of loss of the Sec23 charge interface, we revisited the other interaction sites on Sec23 to test whether combinatorial mutations would similarly impact viability. We first tested the F380L mutation, equivalent to human F382L, which impairs coat assembly (Fromme et al., 2007). As described previously (Fromme et al., 2007), the F380L mutation alone had no impact on viability, even when combined with a *sed4Δ* mutation (Fig. 4 E). Moreover, when F380L was added to the *sec23-Δgel* mutant, cells were still viable (Fig. 4 E). Thus, of the known interactions, abrogation of the charge-based interface has the most profound effect on COPII coat assembly. Our findings reveal that three different modes of interaction drive association between the inner and outer coats: a nucleotide-driven interface mediated by the active fragment, multivalent interaction supported by linear PPP motifs, and a charge-driven interface. Whether these different interactions bind to the same Sec23 molecule or instead bridge adjacent Sec23–Sec24 complexes to contribute to inner coat array formation remains to be determined.

### Cargo contributions to coat assembly and function

**Figure 5.**
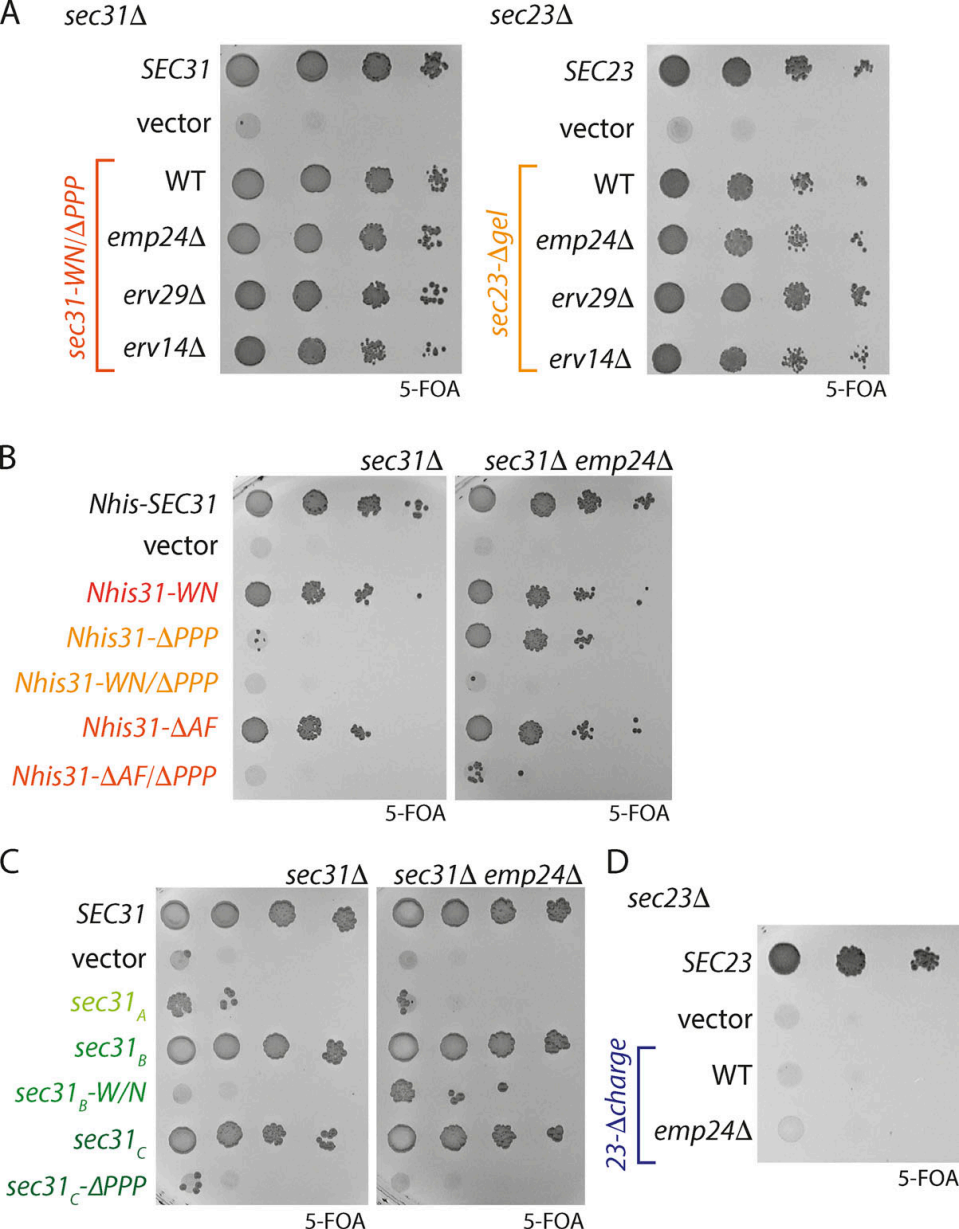
**Cargo receptor mutants influence viability of coat assembly mutants. (A)** *EMP24*, *ERV29*, and *ERV14* were deleted in *sec31Δ* and *sec23Δ* strains and growth of *sec31-WN/ΔPPP* and *sec23-Δgel* tested on media containing 5-FOA. Deletion of cargo receptors had no impact on viability of these interface mutants. **(B)** Growth of each of the *Nhis-Sec31* mutants indicated was tested in *sec31Δ* and *sec31Δ emp24Δ* backgrounds, revealing that loss of Emp24 reversed the lethality associated with *Nhis-sec31-ΔPPP*, but not other mutants. **(C)** The Sec31 fragment mutants indicated were tested in *sec31Δ* and *sec31Δ emp24Δ* backgrounds, revealing no rescue. **(D)** The plasmids indicated were introduced into *sec23Δ* or *sec23Δ emp24Δ* strains and tested for growth on 5-FOA.

Cargo proteins are not inert participants in vesicle formation; rather, they influence coat function in multiple ways. On one hand, cargo proteins are thought to contribute to coat stability by retaining subunits after GTP hydrolysis (Sato and Nakano, 2005; Iwasaki et al., 2017). On the other hand, cargo can alter the local membrane bending energy, thereby imposing a requirement for a robust coat to enforce curvature (Copic et al., 2012; D’Arcangelo et al., 2015; Gómez-Navarro et al., 2020). We therefore tested whether deletion of the major ER export receptors would impact the viability of coat interaction mutants. We deleted *EMP24*, *ERV29*, or *ERV14* in *sec31Δ* and *sec23Δ* backgrounds and first tested for synthetic sick/lethal interactions with coat interface mutants. We reasoned that deleting cargo might further destabilize coat assembly and impair vesicle formation when coat interfaces were weakened. Neither of the viable interface mutants, *sec31-WN/ΔPPP* and *sec23-Δgel*, showed reduced fitness when ER export receptors were abrogated (Fig. 5 A). Although these receptors are among the most abundant constituents of COPII vesicles (Otte et al., 2001; Ho et al., 2018), other essential cargo proteins like SNAREs probably still contribute significant recruitment capacity to stabilize the coat.

We next tested whether cargo depletion might confer phenotypic rescue to coat mutants that are impaired in their assembly. In an *emp24Δ* background, reduced cargo burden creates a permissive condition that allows growth when coat scaffolding is compromised (Copic et al., 2012; D’Arcangelo et al., 2015). We reasoned that when the force required to induce membrane curvature is reduced, weakened coat interfaces might suffice for viability. Indeed, the lethal *Nhis-sec31-ΔPPP* mutant was rescued by deletion of *EMP24* (Fig. 5 B). In contrast, the combinatorial mutants (*Nhis-sec31-WN/ΔPPP* and *Nhis-sec31-ΔAF/ΔPPP*) were not rescued by loss of *EMP24* (Fig. 5 B), suggesting that the simultaneous loss of both interfaces causes an assembly defect too great to be compensated forcargo. Since the *Nhis-sec31* mutants are compromised both for inner/outer coat interfaces and cage vertex formation, the phenotypic rescue in the *emp24Δ* background could be due to reduced scaffolding driven by cage assembly rather than a weakened coat assembly. We therefore tested other interface mutants that are lethal without destabilizing the Sec13–Sec31 cage. The shortened mutants (*sec31_A_* and *sec31_C_-ΔPPP*) were not rescued by *EMP24* deletion, and the *sec31_B_-WN* mutant was only marginally viable in this background (Fig. 5 C). Similarly, the *sec23-Δcharge* mutant remained inviable in a *sec23Δ emp24Δ* strain (Fig. 5 D). Together, these growth phenotypes indicate that deficits in membrane scaffolding can be compensated for by loss of cargo-driven membrane rigidity but that coat assembly interfaces are resistant to such rescue.

### Coat assembly can be driven by diverse sequences with conserved properties

**Figure 6.**
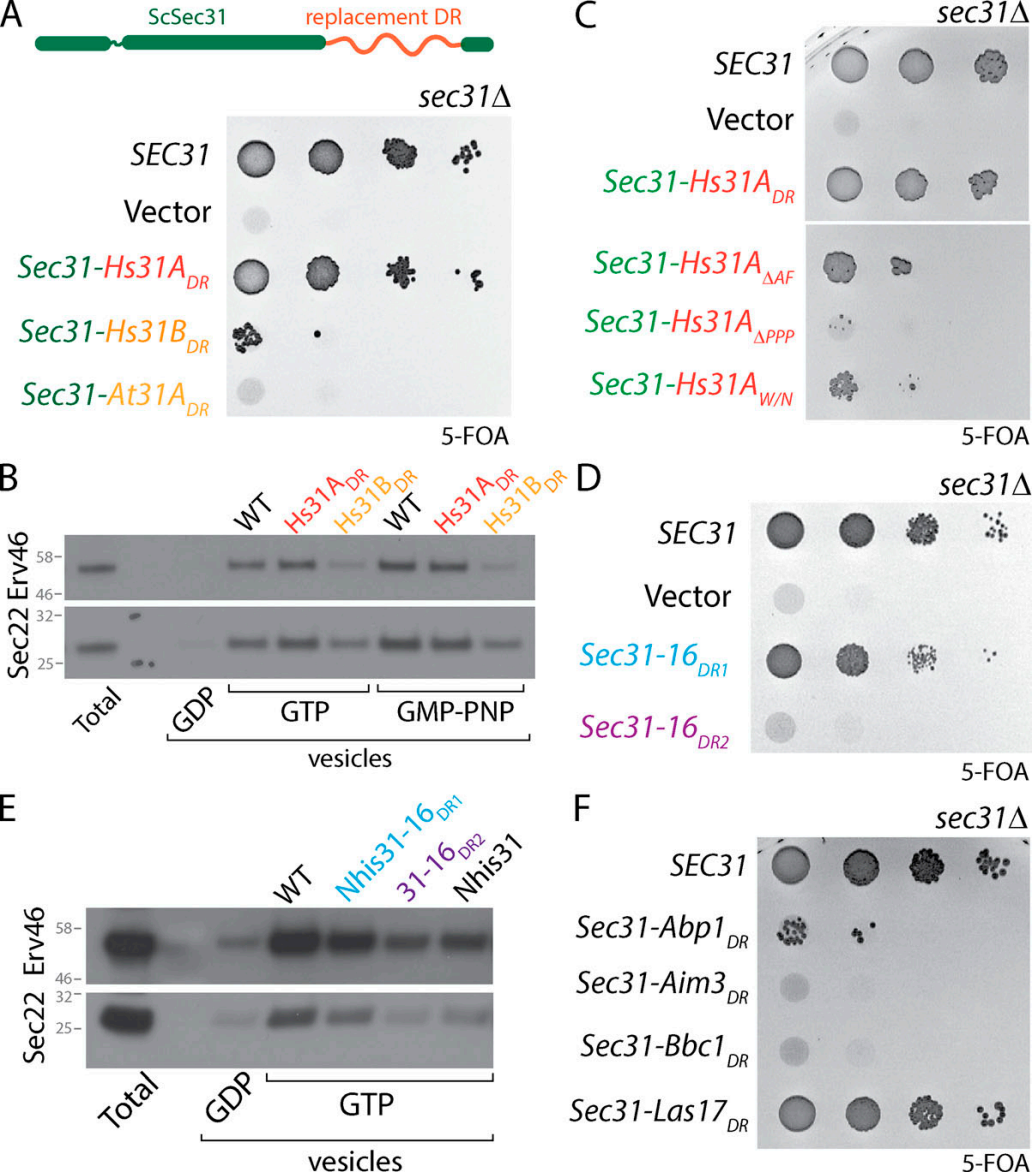
**Sec31 disordered region can be functionally replaced with unrelated sequences. (A)** Serial dilutions of the indicated constructs where the yeast Sec31 disordered region was replaced with that of other species reveals that only the human Sec31A disordered region can support viability. **(B)** In vitro microsomal budding experiments using the indicated proteins correlates with viability; only the Hs31A fusion can support vesicle formation with both GTP and GMP-PNP. **(C)** Serial dilutions of the indicated strains reveals that deletion of the active fragment, or mutation of the PPP or WN motifs within the Hs31A disordered region reduces viability. **(D)** Serial dilutions of Sec31-Sec16 disordered region chimera reveals that only the first Sec16 disordered region can support viability when replacing the Sec31_DR_. **(E)** In vitro budding from microsomal membranes with the indicated proteins correlates with viability, with only Nhis-Sec31-16_DR1_ budding. **(F)** Serial dilutions of chimeric Sec31 constructs containing the indicated unrelated disordered regions reveals that the Las17 domain swap supports viability.

Our bioinformatics analysis of the Sec31 proline-rich disordered region revealed conserved properties across species even though primary sequence similarity was low (Fig. S3, A and B). This is in contrast to sequence conservation across the structured domains, which is relatively high (Fig. S3 B). We reasoned that if coat assembly is driven by combinations of weak interactions rather than specific protein sequence properties, then a disordered region from an orthologous Sec31 might functionally replace the yeast disordered region. We created chimeric proteins where the yeast disordered region was replaced with that of human Sec31A, human Sec31B or *A. thaliana* Sec31A. When these constructs were expressed in yeast, only the chimera with the disordered region of HsSec31A (*sec31-Hs31A_DR_*) supported viability (Fig. 6 A). We tested the two yeast-human chimeric proteins in budding, where activity mirrored the in vivo phenotypes, with only Sec31-Hs31A_DR_ able to drive budding from microsomal membranes (Fig. 6 B). Deletion of the active fragment or mutation of either the PPP motifs or the W/N GTPase stimulatory residues in the yeast–human hybrid construct reduced viability (Fig. 6 C), confirming that these interfaces are a conserved mechanism of driving coat assembly.

**Figure S4.**
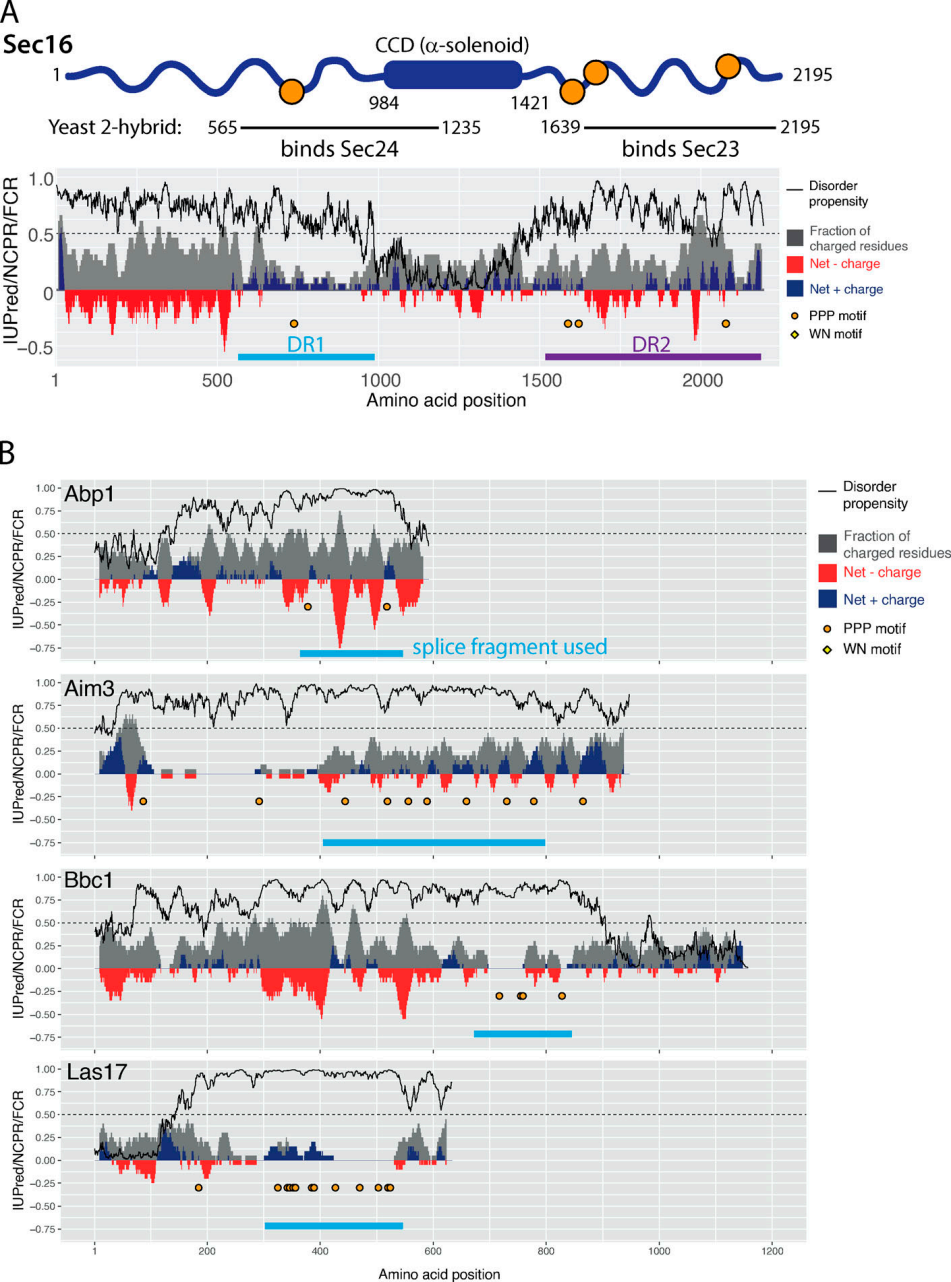
**Charge-disorder analysis for unrelated PPP-containing proteins. (A)** Diagram and charge-disorder plot of Sec16, highlighting the conserved central domain (CCD) and two disordered regions, DR1 (light blue) and DR2 (purple), that interact with Sec24 and Sec23, respectively. Plot is annotated as described in Fig. S2 A. **(B)** Charge/disorder plots for a subset of proteins identified in the yeast genome that contain multiple PPP motifs that were selected for testing as replacements for the Sec31 disordered region. Fragments selected for splicing into Sec31 are indicated by the blue bars.

Given that the human Sec31A disordered region can function in yeast despite divergence in protein sequence (∼30% similarity), we sought to test whether a more distantly related domain could also function in assembly. We first asked whether the disordered regions of Sec16 could replace that of Sec31. Sec16 has two intrinsically disordered regions that lie upstream and downstream of a structured helical domain that interacts with Sec13 (Whittle and Schwartz, 2010; Fig. S4 A). The upstream disordered region (DR1) interacts with Sec24 (Gimeno et al., 1996) and contains a single PPP motif and several positive charge clusters. The downstream disordered region (DR2) interacts with Sec23 (Gimeno et al., 1996) and contains multiple PPP motifs and significant areas of both net positive and negative charge (Fig. S4 A). We replaced the Sec31 disordered region with Sec16_DR1_ or Sec16_DR2_ and tested each chimera for their ability to function in place of Sec31. Sec31-16_DR1_ could complement a *sec31Δ* strain, despite low amino acid similarity (∼35% local pairwise similarity), whereas Sec31-16_DR2_ was not viable (Fig. 6 D). Vesicle formation in vitro mirrored the growth phenotype, with Sec31-16_DR1_ supporting budding and Sec31-16_DR2_ inactive (Fig. 6 E). The in vitro budding experiment used Nhis–Sec31-16_DR1_ because cleavage of the affinity tag used for purification caused the protein to degrade. Nonetheless, Nhis–Sec31-16_DR1_ supported moderate budding with GTP, in contrast to Nhis–Sec31 (Fig. 6 D). This difference likely stems from the inability of the Sec16 disordered region to stimulate GTP hydrolysis, thereby stabilizing coat assembly even in the presence of GTP.

We next sought to further test plasticity of the coat assembly interface by coopting affinity from evolutionarily diverged proteins that share similar properties. We searched the yeast genome for proteins unrelated to the COPII pathway that have predicted disordered regions and multiple PPP motifs. We identified a number of such proteins that participate in endocytosis and actin regulation and in RNA processing. In a recent analysis of yeast disordered segments (Zarin et al., 2019), several of the endocytosis/actin proteins had multiple properties within disordered regions that were similar to Sec31 (Fig. S4 B). We therefore tested whether some of these disordered regions could function in COPII coat assembly when replacing the endogenous Sec31 disordered region. Indeed, the disordered region of Las17 could functionally replace that of Sec31 to support viability (Fig. 6 F). Las17 is an actin assembly factor that uses its proline-rich domain to nucleate actin filaments (Urbanek et al., 2013). This mode of interaction is shared by other components of the actin/endocytic system and has parallels with the COPII coat in that multivalent weak interactions drive assembly (Sun et al., 2017). The Las17 region we tested shares several key features relevant to Sec31 function, including multiple PPP motifs, clusters of net-positive charge in the absence of net negative charge, and significant length. Since Las17 has no active fragment region, clearly these features alone suffice to generate a functional coat, highlighting the plasticity of the inner/outer coat interaction.

## Discussion

Together, our mutagenesis and domain replacement experiments reveal a remarkably plastic mechanism of coat assembly, where multiple independent assembly interfaces mutually stabilize each other. Each individual interaction is dispensable, but in concert, they drive function and stabilize the coat to prevent premature disassembly. Our extensive mutagenesis of Sec31 (Fig. 2) reveals how different assembly elements contribute: the active fragment provides nucleotide-dependent affinity contributed in large part by the W/N motif; linear PPP motifs form weak but specific binding interfaces; a charge-driven interface provides essential affinity; and multivalence of interactions contributes to robust assembly. The ability for loss of one element to be compensated for by another suggests that the different interfaces are partially redundant but reinforce each other to make a robust system. Orthologous Sec31 proteins across species may alter how they employ these interfaces to create functional proteins that might exhibit distinct assembly properties. Our analysis further suggested length effects may also be important, because multivalent PPP-containing stretches of disordered sequence below a certain length threshold were not viable. The precise role of disordered region length remains to be explored; one possibility is that a shortened disordered region (for example the Sec31_E_ fragment, which still contains multiple interaction sites) fails to assemble because of an inability to bridge adjacent Sec23 complexes.

A coat assembly/disassembly mechanism driven by multiple relatively weak interactions provides a possible mechanistic explanation for how a metastable structure like a vesicle coat can form in a controlled manner. By building up from initially weak, transient interactions, the coat can be recruited locally but is not committed to full assembly until a threshold of both inner and outer coat components is reached. Moreover, upstream regulatory factors, like Sec16 and TANGO1, that use the same interactions can prime an exit site for Sec31 recruitment and organize the inner coat. Once Sec31 is engaged, its disordered region can act as molecular velcro, being strong yet readily reversible. Coat propagation could feed forward as cage vertex interactions (mediated by the β-propeller domain of Sec31) bring in additional outer coat rods to in turn organize more inner coat complexes. As the diversity of Sec31 paralogs expands, as seen in multicellular organisms, disordered regions may fuel evolution of altered interfaces that allow for fine-tuning of vesicle assembly, disassembly, and geometry (Babu et al., 2012). For instance, absence of GTP stimulatory residues may yield a more stable coat structure, whereby the GTPase activity of the coat is not accelerated. Such stability may prolong initial events during coat assembly to favor formation of noncanonical carriers (Hutchings and Zanetti, 2019).

The multiple interfaces between Sec23 and Sec31 contribute to persistence of the polymerized coat even after GTP hydrolysis by Sar1 and its release from the membrane. Evidence for preserved coat elements on Sar1-free vesicles comes from immunogold labeling experiments that revealed persistent Sec23 and Sec13 on COPII vesicles generated with GTP (Barlowe et al., 1994). Similarly, multivalent combinatorial interactions provide an excellent mechanism for how a metastable coat polymer can be disassembled without the need for uncoating chaperones that expend energy. A combinatorial binding mode allows a stable structure to “breathe” via dynamic weak individual interactions that permit an opposing similarly weak interaction to compete and destabilize the structure. In the case of the COPII coat, local uncoating might be triggered by Golgi-localized kinases like Hrr25 (Lord et al., 2011) that would reverse a charge-based interaction and help ensure the directionality of vesicle traffic.

Our findings of COPII coat assembly driven by a network of interactions involving physicochemical properties like disorder and net charge, along with multivalent weak motif-driven interactions, provide a blueprint for how intrinsically disordered regions are used for dynamic assembly and disassembly. Each of the major coat complexes have significant disordered regions within their subunits (Pietrosemoli et al., 2013), and the flexible nature of the clathrin light chain is important in coat assembly (Wilbur et al., 2010). Our approach and the findings that we present here provide a conceptual framework to now discover and investigate how such disordered regions of coat complexes evolve to accommodate specific trafficking needs of an organelle, cell, and organism.

## Materials and methods

### Strains and plasmids

Yeast strains used in this study are listed in Table S1. Strains were constructed using standard genetic knockout and LiAc transformation methods. Cultures were grown at 30°C in standard rich medium (YPD: 1% yeast extract, 2% peptone, and 2% glucose) or synthetic complete medium (SC: 0.67% yeast nitrogen base and 2% glucose supplemented with amino acids) as required. For testing viability, strains were grown to saturation in SC medium selecting for the mutant plasmid overnight at 30°C. 10-fold serial dilutions were made in 96-well trays before spotting onto 5-fluoroorotic acid (5-FOA) plates (1.675% yeast nitrogen base, 0.08% complete supplement mixture, 2% glucose, 2% agar, and 0.1% 5-FOA). Plates were scanned at day 2 or 3 after spotting and growth at 30°C.

Plasmids used in this study are listed in Table S2. Standard cloning methods were used, including PCR amplification of yeast genes from genomic DNA, cloning into standard yeast expression vectors (Sikorski and Hieter, 1989), site-directed mutagenesis using the QuikChange system (Agilent), and Gibson Assembly (New England Biolabs) as per manufacturers’ instructions.

### Protein expression and purification

Sar1 was prepared as described (Shimoni and Schekman, 2002). Briefly, GST-Sar1 was expressed in bacterial cells by induction for 2 h with IPTG. Cells were lysed by sonication, and lysates were clarified and incubated with glutathione-agarose beads. The beads were then washed, and GST-free Sar1 was generated by thrombin cleavage.

Sec23–Sec24 (Sec23 and His-Sec24) and Sec13–Sec31 (Sec13 and His-Sec31) complexes were in Sf9 cells using the pFastbac system. 500 ml of protein-expressing cells were collected and washed with PBS before freezing in liquid nitrogen. Cell pellets were lysed with a Dounce homogenizer in cold lysis buffer (20 mM Hepes, pH 8, 250 mM sorbitol, 500 mM KOAc, 1 mM DTT, 10 mM imidazole, and 10% vol/vol glycerol). Lysates were cleared in JA 25–50 rotor (22,000 rpm, 1 h, 4°C), and the supernatant was filtered through a 0.45-µm membrane before loading onto a HisTrap HP column (GE Healthcare). The following steps were done using the ÄKTA purifier system (GE Healthcare), where elution buffer was lysis buffer supplemented with 500 mM imidazole. The column was washed with 4% and 10% elution buffer followed by elution using a linear gradient to 100% elution buffer. Peak fractions were checked by SDS-PAGE followed by Coomassie staining. Verified fractions were mixed in a 3:1 ratio with QA buffer (20 mM Tris, pH 7.5, 1 mM MgOAc, 1 mM DTT, and 10% vol/vol glycerol) and loaded onto a HiTrap Q HP column (GE Healthcare). The protein was eluted using a linear salt gradient to a final concentration of 1 M NaCl. Peak fractions were verified using SDS-PAGE and Coomassie staining and flash-frozen in liquid nitrogen. For removal of the 6xHis tag on Sec31, an overnight cleavage with His-tagged Tobacco Etch Virus Protease was included after the verification of Ni-immobilized metal affinity chromatography fractions. The cleavage was done simultaneously with dialysis into Lysis buffer. Uncleaved protein and His-tagged Tobacco Etch Virus Protease were removed by flowing through the HisTrap HP column before continuing to the ion-exchange step.

### In vitro budding from microsomal membranes

Microsomal membranes were prepared from yeast as described previously (Wuestehube, 1992). Briefly, yeast cells were grown to mid-log phase in YPD (1% yeast extract, 2% peptone, and 2% glucose), harvested and resuspended in 100 mM Tris, pH 9.4/10 mM DTT to 40 OD_600_/ml, and then incubated at room temperature for 10 min. Cells were collected by centrifugation, resuspended to 40 OD_600_/ml in lyticase buffer (0.7 M sorbitol, 0.75× YPD, 10 mM Tris, pH 7.4, and 1 mM DTT + lyticase 2 µl/OD_600_), and then incubated at 30°C for 30 min with gentle agitation. Cells were collected by centrifugation, washed once with 2X JR buffer (0.4 M sorbitol, 100 mM KOAc, 4 mM EDTA, and 40 mM Hepes, pH 7.4) at 100 OD_600_/ml, and then resuspended in 2X JR buffer at 400 OD_600_/ml before freezing at −80°C. Spheroplasts were thawed on ice, and an equal volume of ice cold dH20 added before disruption with a motor-driven Potter Elvehjem homogenizer at 4°C. The homogenate was cleared by low-speed centrifugation and crude membranes collected by centrifugation of the low-speed supernatant at 27,000 ×*g*. The membrane pellet was resuspended in ∼6 ml buffer B88 (20 mM Hepes. pH 6.8, 250 mM sorbitol, 150 mM KOAc, and 5 mM Mg(OAc)_2_) and loaded onto a step sucrose gradient composed of 1 ml 1.5 M sucrose in B88 and 1 ml 1.2 M sucrose in B88. Gradients were subjected to ultracentrifugation at 190,000 ×*g* for 1 h at 4°C. Microsomal membranes were collected from the 1.2 M/1.5 M sucrose interface, diluted 10-fold in B88, and collected by centrifugation at 27,000 ×*g*. The microsomal pellet was resuspended in a small volume of B88 and aliquoted in 1 mg total protein aliquots until use.

Budding reactions were performed as described previously (Miller et al., 2002). Briefly, 1 mg of microsomal membranes per six to eight reactions was washed three times with 2.5 M urea in B88 and three times with B88. Budding reactions were set up in B88 to a final volume of 250 µl at the following concentrations: 10 µg/µl Sar1, 10 µg/µl Sec23–Sec24, 20 µg/µl Sec13–Sec31, and 0.1 mM nucleotide. Where appropriate, an ATP regeneration mix was included (final concentration: 1 mM ATP, 50 µM GDP-mannose, 40 mM creatine phosphate, and 200 µg/ml creatine phosphokinase). Reactions were incubated for 30 min at 25°C and a 12-µl aliquot collected as the total fraction. The vesicle-containing supernatant was collected after pelleting the donor membrane (15,000 rpm, 2 min, 4°C). Vesicle fractions were then collected by centrifugation in a Beckman TLA-55 rotor (50,000 rpm, 25 min, 4°C). The supernatant was aspirated, and the pelleted vesicles were resuspended in SDS sample buffer and heated for 10 min at 55°C with mixing. The samples were then analyzed by SDS-PAGE and immunoblotting for Sec22 (Miller laboratory antibody) and Erv46 (a gift from Charles Barlowe, Dartmouth College, Hanover, NH). Budding reactions were performed at least three times for each mutant, and results were consistent across replicates. One representative example is shown.

### In vitro binding to small synthetic liposomes

Liposome binding experiments were performed as described previously (Miller et al., 2002). Briefly, synthetic liposomes of “major/minor” composition (50 mol% phosphatidylcholine, 21 mol% phosphatidylethanolamine, 8 mol% phosphatidylserine, 5 mol% phosphatidic acid, 9 mol% phosphatidylinositol, 2.2 mol% phosphatidylinositol-4-phosphate, 0.8% mol% phosphatidylinositol-4,5-bisphosphate, and 2 mol% cytidine-diphosphate-diacylglycerol, supplemented with 2 mol% Texas red–phosphatidylethanolamine and 20% [wt/wt] ergosterol) were dried to a lipid film in a rotary evaporator and rehydrated in HKM buffer (20 mM HSepes, pH 7.0, 160 mM KOAc, and 1 mM MgCl_2_). The lipid suspension was extruded 17 times through a polycarbonate filter of 0.4-µm pore size. Purified COPII components and lipids were mixed to final concentrations of 0.27 mM liposomes, 15 µg/ml Sar1, 20 µg/ml Sec23–Sec24, 30 µg/ml Sec13–Sec31, and 0.1 mM nucleotide in 75 µl HKM buffer. Binding reactions were incubated for 30 min at 25°C. Each sample was mixed with 50 µl 2.5 M sucrose-HKM, and 100 µl was then transferred to an ultracentrifuge tube, overlaid with 100 µl 0.75 M sucrose-HKM, and 20 µl HKM. The gradients were spun (100,000 rpm, 25 min, 24°C with slow acceleration/deceleration) in a Beckman TLA-100 rotor. The top 30 µl of the gradients was collected and normalized for lipid recovery using a Typhoon FLA 7000 scanner (GE Healthcare). Samples were then resolved by SDS-PAGE and visualized using SYPRO red staining. Binding reactions were performed at least three times for each mutant. One representative example is shown, and bands were quantified using Fiji software, with statistical analysis performed in Prism GraphPad.

### GUV budding and electron microscopy

GUVs of major/minor composition were made by electroformation, as described previously (Hutchings et al., 2018). Purified COPII proteins (1 µM Sar1, 320 nM Sec23–Sec24, and 170 nM Sec13–Sec31) were incubated with GUVs (10% vol/vol), 1 mM GMP-PNP (Sigma), and 2.5 mM EDTA in HKM buffer (20 mM Hepes, 50 mM KOAc, and 1.2 mM MgCl_2_, pH 6.8) at room temperature for 1–2 h. Mutants were used in some reactions, as indicated. For negative-stain electron microscopy, 4 µl of the reconstitution was added to negatively glow-discharged grids (EM Sciences; CF300-Cu) and stained with 2% uranyl acetate following standard procedures. Images were recorded on Tecnai electron microscopes operated either at 200 kV and equipped with a DE20 (Direct Electron) detector or at 120 kV and equipped with a charge-coupled device camera (Gatan). Images were collected with doses of 20–30 e^−^/pixel/second.

### Bioinformatic analyses

For analyses of sequence features of disordered regions, sequences of *S. cerevisiae* Sec31, Sec16, Ab1p, Aim3, Bbc1, and Las17, human Sec31A and Sec31B, and *A*.* thaliana *Sec31A were collected from the UniProt database. The residue-specific disorder propensity was calculated using IUPred2A using long disorder mode (Mészáros et al., 2018). Charge properties, including fraction of charged residues and net charge per residue, were calculated using package localCIDER (Holehouse et al., 2017), with a sliding window size of 20. PPP motifs are identified as PPP_n_ (*n* > 2), PPAP, or PAPP. The analyses were performed in Python. Data were assembled and plotted by R using custom-written scripts. For sequence similarities between disordered regions of Sec31 orthologues, yeast Sec31 (749–1,174), human Sec31A (780–1,112), human Sec31B (811–1,078), and *A*. *thaliana *Sec31A (722–860) were chosen for analysis. Each sequence was further divided into three parts: an IDR, an N-terminal structured region, and a C-terminal structured region, with the IDR in each sequence separating the N-terminal structured region and C-terminal structured region. The residues of the IDR in each orthologue are defined as yeast Sec31 (764–1,174), human Sec31A (800–1,091), human Sec31B (793–1,053), and *A*.* thaliana *Sec31A (719–870). Disordered regions including Abp1 (361–528), Aim3 (401–788), Bbc1 (670–843), and Las17 (306–529) were also chosen to compare similarity. These residues were predicted to have high disorder propensity (IUPred >0.5) and chosen to match the experimental constructs. Due to the striking difference among the lengths of the IDRs, pairwise local alignment was used to retrieve pairwise sequence similarity between the yeast Sec31 regions and corresponding structured or disordered regions. Sequence alignments were performed using EMBOSS water with default parameters (Madeira et al., 2019).

### Data and materials availability

All data are available in the article or supplemental material; plasmids and strains described can be obtained from E.A. Miller.

### Online supplemental material

Fig. S1 shows the structure of Sec13–Sec31, electron microscopy of GUV experiments with WT and mutant Sec23 and Sec31, and control experiments that demonstrate membrane tabulation requires the full COPII coat. Fig. S2 shows quantification of protein recruitment in liposome binding assays. Fig. S3 shows charge/disorder plots for Sec31 orthologues from different species and a protein similarity comparison for those proteins. Fig. S4 shows charge/disorder plots for Sec16 and other unrelated PPP-containing proteins used in complementation experiments shown in Fig. 6. Table S1 describes the yeast strains used in this study. Table S2 describes the plasmids used in this study.

## Supplementary Material

Table S1 describes the yeast strains used in this study.

Table S2 describes the plasmids used in this study.
