# Supplementary material for: Combinatorial multivalent interactions drive cooperative assembly of the COPII coat
Source: J Cell Biol. 2020 Sep 30;219(11):e202007135. doi: 10.1083/jcb.202007135 (PMC7594496; doi:10.1083/jcb.202007135)
Supplement: Table S1 — describes the yeast strains used in this study. [file JCB_202007135_TableS1.docx]

**Table S1 – Yeast strains**

| **Strain** | **Genotype** | **Source** |
| --- | --- | --- |
| LMY570 | *sec23::HIS3 ade2-101oc leu2-Δ1 lys2-801am trp1-Δ63 +[pRS416::SEC23-URA3]* | Miller Lab |
| LMY868 | *sec23::HIS3 sed4::KANMX ade2-101oc leu2-Δ1lys2-801am trp1-Δ63 +[pRS416::SEC23-URA3]* | Miller Lab |
| LMY1249 | *sec31::NAT pep4::TRP ade2-1 his3-11 leu2-3,112 + [pYCp50::SEC31-URA3]* | (Hutchings et al., 2018) |
| VSY015 | *sec31::NAT emp24::KANMX pep4::TRP ade2-1 his3-11 leu2-3,112 + [pYCp50::SEC31-URA3]* | This study |
| VSY016 | *sec31::NAT sed4::KANMX pep4::TRP ade2-1 his3-11 leu2-3,112 + [pYCp50::SEC31-URA3]* | This study |
| NGY198 | *sec31::TRP1 erv29::HIS3 ade2-1 leu2,3-112 trp1-1 [pRS316::SEC31]* | This study |
| NGY291 | *sec31::TRP1 erv14::KANMX ade2-1 leu2,3-112 trp1-1 [pRS316::SEC31]* | This study |
| NGY200 | *sec23::HIS3 erv29::LEU2 ade2-101oc lys2-801am trp1-Δ63 +[pRS416::SEC23-URA3]* | This study |
| NGY553 | *sec23::HIS3 erv14::KANMX ade2-101oc leu2-Δ1 lys2-801am trp1-Δ63 +[pRS416::SEC23-URA3]* | This study |
| VSY017 | *sec23::HIS3 emp24::KANMX ade2-101oc leu2-Δ1 lys2-801am trp1-Δ63 +[pRS416::SEC23-URA3]* | This study |
|  |  |  |
