# Supplementary material for: Combinatorial multivalent interactions drive cooperative assembly of the COPII coat
Source: J Cell Biol. 2020 Sep 30;219(11):e202007135. doi: 10.1083/jcb.202007135 (PMC7594496; doi:10.1083/jcb.202007135)
Supplement: Table S2 — describes the plasmids used in this study. [file JCB_202007135_TableS2.docx]

**Table S2 – Plasmids**

| **Name** | **Description** | **Source** |
| --- | --- | --- |
| RSB600 | pRS313 (HIS3, CEN) | (Sikorski and Hieter, 1989) |
| RSB602 | pRS314 (TRP1, CEN) | (Sikorski and Hieter, 1989) |
| RSB604 | pRS315 (LEU2, CEN) | (Sikorski and Hieter, 1989) |
| RSB606 | pRS316 (URA3, CEN) | (Sikorski and Hieter, 1989) |
| pLK140 | *pRS314 (TRP1::LEU2) SEC23* under native promoter and CYC1 terminator | Miller Lab |
| LMB352 | *pRS414 (TRP1, CEN)* | (Sikorski and Hieter, 1989) |
| LMB353 | *pRS414 with SEC23 under native promoter* | (Fromme et al., 2007) |
| VSB44 | *sec23-Δgel* (Y678-Y684 replaced with SGS) mutation in pLK140 | This study |
| VSB81 | *sec23-Δcharge* (D2R/E4R/D8R/D44R/E45R/E392R/E393R/E526R) mutation in pLK140 | This study |
| VSB164 | *sec23-Δgel* LEU::TRP marker swapped VSB44 | This study |
| VSB165 | *sec23-F380L* mutation in LMB353 | This study |
| VSB166 | *sec23-F380L* mutation in VSB164 | This study |
| pAC20 | *pRS316(URA3::LEU2*) genomic fragment of *SEC31* with 6xHis encoding sequence inserted after M1 | Miller Lab |
| VSB50 | sec31-W922A/N923A mutation in pAC20 | This study |
| VSB69 | *sec31-ΔPPP* (PPP853-855SGS/PPPP965-968GSGS/PPP981-983GSG/APPP1041-1044GSGS/PPAP1057-1060GSGS/PPP1096-1098GSG/PPP1107-1109SGS) mutation in pAC20 | This study |
| VSB87 | sec31-W922A/N923A mutation in VSB69 | This study |
| VSB160 | sec31-ΔAF (ΔP899-N945) mutation in pAC20 | This study |
| VSB161 | sec31-ΔAF (ΔP899-N945) mutation in VSB69 | This study |
| VSB49 | *pRS313 (HIS3, CEN) SEC31* with native 500 bp upstream and downstream of gene cloned in BamHI/NotI | This study |
| VSB58 | *sec31-ΔPPP* (PPP853-855SGS/PPPP965-968GSGS/PPP981-983GSG/APPP1041-1044GSGS/PPAP1057-1060GSGS/PPP1096-1098GSG/PPP1107-1109SGS) mutation in VSB49 | This study |
| VSB59 | sec31-W922A/N923A mutation in VSB58 | This study |
| VSB119 | *sec31-ΔAF* (ΔP899-N945) mutation in VSB49 | This study |
| VSB157 | *sec31-ΔAF/ΔPPP* (ΔP899-N945) mutation in VSB58 | This study |
| VSB60 | *sec31A* (ΔI881-K1114) mutation in VSB49 | This study |
| VSB61 | *sec31B* (ΔT764-P880/ΔE998-K1114) mutation in VSB49 | This study |
| VSB62 | *sec31C* (ΔT764-S997) mutation in VSB49 | This study |
| VSB63 | *sec31D* (ΔT764-S974/ΔP1051-K1114) mutation in VSB49 | This study |
| VSB64 | *sec31E* (ΔT764-T1050) mutation in VSB49 | This study |
| VSB149 | *sec31B-W/N* (W922A/N923A) mutation in VSB61 | This study |
| VSB150 | *sec31B-ΔPPP1* (PPPP965-968GSGS) mutation in VSB61 | This study |
| VSB151 | *sec31B-ΔPPP2* (PPP981-983GSG) mutation in VSB61 | This study |
| VSB152 | *sec31B-ΔPPP1,2* (PPPP965-968GSGS/PPP981-983GSG) mutation in VSB61 | This study |
| VSB155 | *sec31B-W/N/ΔPPP1,2* (W922A/N923A/PPPP965-968GSGS/PPP981-983GSG) mutation in VSB61 | This study |
| VSB156 | *sec31C-Δ3PPP* (APPP1041-1044GSGS/PPAP1057-1060GSGS/PPP1107-1109SGS) mutation in VSB62 | This study |
| VSB89 | *sec31C-Δ4PPP* (APPP1041-1044GSGS/PPAP1057-1060GSGS/PPP1096-1098GSG/PPP1107-1109SGS) mutation in VSB62 | This study |
| VSB70 | *sec31-Hs31ADR* (T764-K1114 replaced with HsSec31A P800-P1091) mutation in VSB49 | This study |
| VSB71 | *sec31-Hs31BDR* (T764-K1114 replaced with HsSec31B S793-E1053) mutation in VSB49 | This study |
| VSB72 | *sec31-At31ADR* (T764-K1114 replaced with AtSec31A A719-L870) mutation in VSB49 | This study |
| VSB90 | *sec31-Hs31ADR-ΔPPP* (PPPP838-841SGSG/APP900-902GSG/PPPP944-947SGSG/APP960-962GSG/PP970-971GS/PPA998-1000SGS) mutation in VSB69 | This study |
| VSB162 | *sec31-Hs31ADR-ΔAF* (ΔA980-M1015) mutation in VSB70 | This study |
| VSB163 | *sec31-Hs31ADR-W/N* (W995A/N996A) mutation in VSB70 | This study |
| VSB73 | *sec31-Abp1DR* (T764-K1114 replaced with Abp1 G361-E528) mutation in VSB49 | This study |
| VSB74 | *sec31-Aim3DR* (T764-K1114 replaced with Aim3 I401-N788 mutation in VSB49 | This study |
| VSB75 | *sec31-Bbc1DR* (T764-K1114 replaced with Bbc1 S670-S843) mutation in VSB49 | This study |
| VSB76 | *sec31-Las17DR* (T764-K1114 replaced with Las17 I306-P529) mutation in VSB49 | This study |
| VSB82 | *sec31-16DR1* (T764-K1114 replaced with Sec16 G565-D984) mutation in VSB49 | This study |
| VSB83 | *sec31-16DR2* (T764-K1114 replaced with Sec16 Q1513-Q2195) mutation in VSB49 | This study |
| pFB23 | pFastBacHTb-Sec23p | Zanetti Lab |
| pFB24N | pFastBacHTb-Sec24p-NHis | Zanetti Lab |
| pFB13 | pFastBacHTb-Sec13p | Zanetti Lab |
| pFB31N | pFastBacHTb-Sec31p-NHis | Zanetti Lab |
| VSB95 | *sec23-Δgel* (Y678-Y684 replaced with SGS) mutation in pFB23 | This study |
| VSB96 | *sec23-Δcharge* (D2R/E4R/D8R/D44R/E45R/E392R/E393R/E526R) mutation in pFB23 | This study |
| VSB98 | *sec31-ΔPPP* (PPP853-855SGS/PPPP965-968GSGS/PPP981-983GSG/APPP1041-1044GSGS/PPAP1057-1060GSGS/PPP1096-1098GSG/PPP1107-1109SGS) mutation in pFB31N | This study |
| VSB100 | *sec31A* (ΔI881-K1114) mutation in pFB31N | This study |
| VSB101 | *sec31B* (ΔT764-P880/ΔE998-K1114) mutation in pFB31N | This study |
| VSB102 | *sec31C* (ΔT764-S997) mutation in pFB31N | This study |
| VSB103 | *sec31D* (ΔT764-S974/ΔP1051-K1114) mutation in pFB31N | This study |
| VSB104 | *sec31E* (ΔT764-T1050) mutation in pFB31N | This study |
| VSB105 | *sec31-Hs31ADR* (T764-K1114 replaced with HsSec31A P800-P1091) mutation in pFB31N | This study |
| VSB106 | *sec31-Hs31BDR* (T764-K1114 replaced with HsSec31B S793-E1053) mutation in pFB31N | This study |
| VSB107 | *sec31-16DR1* (T764-K1114 replaced with Sec16 G565-D984) mutation in pFB31N | This study |
| VSB108 | *sec31-16DR2* (T764-K1114 replaced with Sec16 Q1513-Q2195) mutation in pFB31N | This study |
